# Aquaculture Reuse Water, Genetic Line, and Vaccination Affect Rainbow Trout (*Oncorhynchus mykiss*) Disease Susceptibility and Infection Dynamics

**DOI:** 10.3389/fimmu.2021.721048

**Published:** 2021-09-22

**Authors:** Jeremy L. Everson, Darbi R. Jones, Amy K. Taylor, Barb J. Rutan, Timothy D. Leeds, Kate E. Langwig, Andrew R. Wargo, Gregory D. Wiens

**Affiliations:** ^1^National Center for Cool and Cold Water Aquaculture, Agricultural Research Service, United States Department of Agriculture (USDA), Kearneysville, WV, United States; ^2^Virginia Institute of Marine Science, William & Mary, Gloucester Point, VA, United States; ^3^Department of Biological Sciences, Virginia Tech, Blacksburg, VA, United States

**Keywords:** rainbow trout, DNA vaccination, infectious hematopoietic necrosis virus, *Flavobacterium psychrophilum*, genetic resistance, natural exposure, reuse water, aquaculture

## Abstract

Infectious hematopoietic necrosis virus (IHNV) and *Flavobacterium psychrophilum* are major pathogens of farmed rainbow trout. Improved control strategies are desired but the influence of on-farm environmental factors that lead to disease outbreaks remain poorly understood. Water reuse is an important environmental factor affecting disease. Prior studies have established a replicated outdoor-tank system capable of varying the exposure to reuse water by controlling water flow from commercial trout production raceways. The goal of this research was to evaluate the effect of constant or pulsed reuse water exposure on survival, pathogen prevalence, and pathogen load. Herein, we compared two commercial lines of rainbow trout, Clear Springs Food (CSF) and Troutex (Tx) that were either vaccinated against IHNV with a DNA vaccine or sham vaccinated. Over a 27-day experimental period in constant reuse water, all fish from both lines and treatments, died while mortality in control fish in spring water was <1%. Water reuse exposure, genetic line, vaccination, and the interaction between genetic line and water exposure affected survival (*P*<0.05). Compared to all other water sources, fish exposed to constant reuse water had 46- to 710-fold greater risk of death (*P*<0.0001). Tx fish had a 2.7-fold greater risk of death compared to CSF fish in constant reuse water (*P ≤* 0.001), while risk of death did not differ in spring water (*P*=0.98). Sham-vaccinated fish had 2.1-fold greater risk of death compared to vaccinated fish (*P*=0.02). Both IHNV prevalence and load were lower in vaccinated fish compared to sham-vaccinated fish, and unexpectedly, *F. psychrophilum* load associated with fin/gill tissues from live-sampled fish was lower in vaccinated fish compared to sham-vaccinated fish. As a result, up to forty-five percent of unvaccinated fish were naturally co-infected with *F. psychrophilum* and IHNV and the coinfected fish exhibited the highest IHNV loads. Under laboratory challenge conditions, co-infection with *F. psychrophilum* and IHNV overwhelmed IHNV vaccine-induced protection. In summary, we demonstrate that exposure to reuse water or multi-pathogen challenge can initiate complex disease dynamics that can overwhelm both vaccination and host genetic resistance.

## 1 Introduction

Infectious diseases are an inherent and important component of both wild fish ecosystems and captive fish populations that include aquaculture (1). Rainbow trout (*Oncorhynchus mykiss*) is a widely studied model fish species (2) with substantial farm value in the US and worldwide (3). Infectious diseases cause appreciable losses in rainbow trout aquaculture and a recent survey of US trout producers indicate 29.1 million fish were lost in 2018 and that infectious disease accounted for 92% of these losses (4). Two frequent pathogens of rainbow trout are infectious hematopoietic necrosis virus (IHNV) [reviewed in (5, 6)] and *Flavobacterium psychrophilum* (*Fp*), the causative agent of bacterial cold water disease (BCWD) [reviewed in (7–10)]. These pathogens can co-occur at farm sites, although the natural co-infection rate of individual fish is unclear. Under laboratory conditions, reciprocal low dose challenge with both pathogens resulted in high mortality, reduced time-to-death, and exacerbated clinical signs (11) consistent with farm site observations of high mortality when both pathogens are present.

Efforts to control these diseases on-farm include both vaccination and selective breeding for increased disease resistance. There is an effective DNA vaccine against IHNV (6, 12–14) as well as experimental vaccines against *F. psychrophilum* (15–17). Efforts to genetically improve disease resistance (defined as enhanced survival following laboratory exposure) in rainbow trout populations are ongoing for both pathogens (18–23). Selective breeding of rainbow trout for resistance to IHNV has been implemented at Clear Springs Foods (CSF) Inc. since the year 2000 as part of a multi-trait selection program (22), resulting in production of the CSF fish line. Based on analysis of laboratory challenge data, selection differential for resistance to IHNV has averaged 10% for eight generations (between yrs. 2000 and 2016) and the mortality rate in challenge trials decreased by an average of 3% per generation (23). However, the genetic stock of this breeding program is not a closed population as there is routine introgression that includes germplasm from the USDA/ARS breeding program located at the Hagerman Fish Culture Experimental Station, Idaho, USA (24). A separate genetic line, the Troutex (Tx) line, has undergone selection for a number of years in Denmark and consists of 5 mating groups with ongoing breeding objectives to increase growth, body shape and yield, and improved survivability/robustness under farm conditions (25). To our knowledge, there has not been a direct comparison of disease resistance or vaccine response between these two lines.

Infectious disease outbreaks are influenced by a myriad of environmental factors including water temperature and quality (1). At many rainbow trout aquaculture facilities, water is serially reused multiple times and is partially reoxygenated by cascade waterfalls between raceway units (26). However, even with this added oxygen, water quality diminishes (e.g. increase in total dissolved solids, turbidity, and fish catabolites) between the serial reuse units resulting in slower growth and reduced survival that cannot be fully reversed by adding supplemental oxygen (27). Exposure to reuse water is associated with an increased stress response and altered gene expression in rainbow trout (28). The impact of water reuse on disease susceptibility is often ignored and most laboratory studies are conducted in first use water with a single pathogen. In contrast, aquaculture conditions can include variable exposure to multiple pathogens under varying environmental conditions (29). How reuse water impacts vaccination and genetic resistance is unknown.

Herein, we test the hypothesis that exposure to reuse water would increase overall disease susceptibility and that this may be affected by trout genetic background and prior vaccination. We exposed two genetic lines, CSF and Tx, that had been either vaccinated or sham vaccinated against IHNV, to three water exposure conditions. These included spring water only (control), a 24 h pulse with reuse water, and finally, a constant exposure to reuse water for a 27-day experimental study period. We also investigate the genetic resistance of each fish-line under defined laboratory conditions by challenge with either IHNV, *F. psychrophilum* or both pathogens in combination. This study reports the first quantification of multi-pathogen infection dynamics under water reuse that is relevant to rainbow trout production.

## 2 Methods

### 2.1 Rainbow Trout Populations and Vaccination

Two all-female rainbow trout lines were evaluated in this study; a Clear Springs Foods production population from Soda Springs, ID, and Troutex production population (ApS, Denmark). We refer to these as CSF- and Tx-lines for convenience with the understanding that these are not closed breeding populations. Two commercial egg lots from winter spawning broodfish were age-matched for hatching date and subsequently reared at the Clear Springs Foods Research facility. Animal procedures at CSF were approved and performed under the guidelines of the University of Idaho IACUC. A subset of each egg lot was also shipped to the Virginia Institute of Marine Science (VIMS), Gloucester Point VA, USA for phenotypic evaluation and is described further under section *2.8 Laboratory challenge*. After hatching and swim-up, fish were fed daily a standard rainbow trout fishmeal-based diet (45% protein: 20% fat; CSF, Inc.) using a standard feed schedule. Both the farm site and CSF Research Facility are supplied by spring water from the Snake River Plain Aquifer. Prior to vaccination, each of the lines were equally distributed across six, 945L tanks that were randomly located in the rearing facility. At the time of vaccination, the CSF-line mean body weight was 13.5g and the Tx-line was 10.8g. Fish were anesthetized by immersion in 100 mg ethyl 3-aminobenzoate methanesulfonate (MS-222, Sigma) with 300 mg sodium bicarbonate per 1L of water. Fish from three tanks per population were vaccinated by intramuscular injection using a 26 g needle with 0.05µg IHNV G protein DNA vaccine pWg (30) in a volume of 25µl sterile PBS and the other three tanks vaccinated with 25µl sterile PBS as a sham vaccination procedure control. At 34 days post-vaccination (~490 temperature degree days), vaccinated and unvaccinated control groups were transferred from the CSF Research facility to an outdoor tank system that has been previously described (28). At the time of transfer, the CSF line averaged 39 ± 3g (mean ± SD of 3 pools of 10 fish) and the Tx line averaged 35 ± 3g and fish were randomly assigned to tanks by treatment.

### 2.2 Experimental Design of Field Experiment and Fish Exposure to Reuse Water

**Figure 1:**
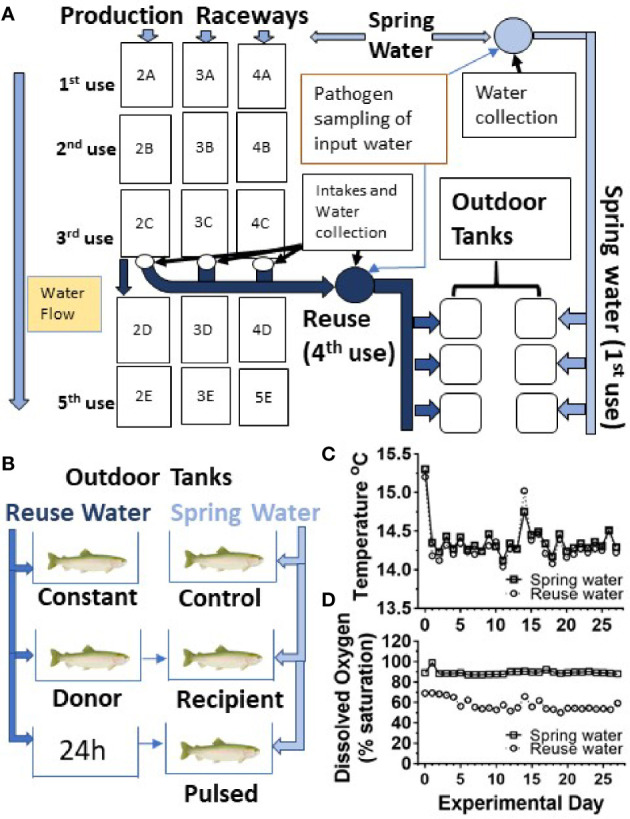
Experimental design and water quality measurements. **(A)** Schematic diagram of water flow to production raceway series 2, 3 and 4 and the collection of first and fourth use water for outdoor tank system. The reuse water has passed through three raceways (3 uses) and was equally comingled prior to supplying the outdoor experimental tank systems, constituting the reuse (4^th^ use) treatment. Water exposure groups **(B)**, temperature **(C)** and oxygen profiles **(D)**. Spring water (squares) Reuse water (circles).

In order to determine the effect of water reuse on infection and survival, fish were transferred from the CSF Research facility to an outdoor, experimental tank system that encompassed twenty, 450 L tanks supplied by water from active raceways containing production fish at a farm site as shown schematically (**Figure 1A**). Fish were moved to the experimental tank system grouped by fish line and vaccine treatment. Fish were netted with a nylon net and 50 fish were counted and placed in two 38L transport tanks containing 19L water and supplied with supplemental oxygen. In this study, twelve tanks received spring water (also referred to as first use water) and eight tanks received water collected after passage through three series of production raceways designated 2A-2C, 3A-3C and 4A-4C series (**Figure 1A**). Throughout, we refer to the fourth use of this water as “reuse” water. Each 450L tank received water at a flow rate of 22.7L per minute, resulting in 3 volume turnovers per hour, which matched the larger production raceways.

Five different water exposure treatments were applied to the experimental tank system and included: Control, Constant, Donor, Recipient, and Pulsed (**Figure 1B**). All fish were initially stocked on “day -1” of the experiment. The Control group was maintained in spring water while the Constant group was maintained in reuse water throughout the experiment. The Donor group was maintained on reuse water but initially housed 120 fish/tank, 20 of which were netted and transferred to the Recipient group after 24 h. The remainder of the Donor group were equivalent to the Constant group as they were maintained on reuse water. The Recipient group was on first use water throughout the experiment with an initial number of 80 fish per tank but after transfer of the 20 fish from the Donor group, matched the final 100 fish/tank of the other groups. The Pulsed group was exposed to reuse water for 24h and then netted and transferred to tanks receiving spring water. Each water treatment included both the CSF and Tx genetic lines that had either been vaccinated (Vac) or sham vaccinated (Shm) (**Table 1**). The initial density per tank was approximately 8.8 kg fish per m^3^ of water, which is about 3- to 10-fold lower than typical fourth-use raceway density. Water quality was measured daily with a YSI Model 6920 V2 water quality sonde equipped with temperature and DO sensors (YSI, Inc., Yellow Springs, OH, USA). Treatments were randomly assigned to tanks within a water system with the exception that reuse water pulsed tanks were immediately adjacent to first use tanks to facilitate transfer.

**Table 1:** Mortality, sampling, and survival proportion of fish in 28-day field experiment (including day -1).

Water Exposure	Genetic line	Treatment	TotalFish	Deaths	Live Sampled(day 1-26)(Censored)	Alive(day 27)	SurvivalProportions(%)^a^	MedianSurvival (days)	Live sampled^b^ (day)
Control	CSF	Shm	97	0	51	46	100.0	ND	1,3,6,10,12,17,19,24,26,27
		Vacc	99	0	51	48	100.0	ND	1,3,6,10,12,17,19,24,26,27
	Tx	Shm	100	0	51	49	100.0	ND	1,3,6,10,12,17,19,24,26,27
		Vacc	100	1	51	48	98.6	ND	1,3,6,10,12,17,19,24,26,27
Constant	CSF	Shm	99	67	32	0	0.0	11	1,3,6,10,12,24
		Vacc	97	61	36	0	0.0	17	1,3,6,10,12,19
	Tx	Shm	100	69	31	0	0.0	9	1,3,6,10,12
		Vacc	98	67	31	0	0.0	9	1,3,6,10,12
Donor	CSF	Shm	99	67	32	0	0.0	16	1,3,6,10,12,24
		Vacc	98	67	31	0	0.0	12	1,3,6,10,12
	Tx	Shm	99	68	31	0	0.0	9	1,3,6,10,12
		Vacc	100	69	31	0	0.0	10	1,3,6,10,12
Pulsed	CSF	Shm	98	4	51	43	93.5	ND	1,3,6,10,12,17,19,24,26,27
		Vacc	100	0	51	49	100.0	ND	1,3,6,10,12,17,19,24,26,27
	Tx	Shm	100	21	51	28	67.5	ND	1,3,6,10,12,17,19,24,26,27
		Vacc	96	7	51	38	88.8	ND	1,3,6,10,12,17,19,24,26,27
Recipient	CSF	Shm	100	6	51	43	89.8	ND	1,3,6,10,12,17,19,24,26,27
		Vacc	100	5	51	44	90.6	ND	1,3,6,10,12,17,19,24,26,27
	Tx	Shm	101	14	51	36	80.4	ND	1,3,6,10,12,17,19,24,26,27
		Vacc	94	1	51	42	97.9	ND	1,3,6,10,12,17,19,24,26,27

a Survival fractions were calculated using the product limit (Kaplan-Meier) method (GraphPad Prism 7.04).

b Seven fish were sampled on days 1, 3, and 6, while 5 or fewer were sampled at remaining time points. ND, Not Determined.

### 2.3 Mortality in Production Raceways That Supplied Reuse Water to Field Experiment

The production raceways supplying water into the experimental tanks were monitored daily as part of ongoing aquaculture operations at the farm site. Mortalities in each production raceway were recorded and removed by farm personnel. At the start of the experiment (day -1) the total fish number in raceway series 2 was estimated at 242,710 (2A=179,212, 2B=32,894, and 2C=30,604 fish, respectively). The total fish number estimated in raceway series 3 was 163,485 (3A=101,429, 3B =33,942 and 3C =28,114 fish, respectively). The total fish number in raceway series 4 was 200,582 (4A= 132,500, 4B =34,451 and 4C= 33,631 fish, respectively). In raceway 3A, on experimental day 7, all fish were moved to another raceway no longer in series with experimental tanks and on day 18, a new lot of 276,283 fish were moved into the raceway. In the series 3 raceways, elevated mortality was ongoing at the initiation of the experiment and subjected to standard farm-site diagnostic procedures for identification of viral and bacterial pathogens. Fish in the raceways and outdoor experimental tanks were fed an in-house produced rainbow trout feed (45% protein and 20% fat, CSF Inc.). The outdoor experimental tanks were fed a 5% feed ration based on the move-in weights of each genetic line and exposed to a natural photoperiod. Fish were fed using 12 h automatic, clockwork belt feeders (Dynamic Aqua-Supply, Ltd., Surrey, BC, Canada). The daily ration was spread evenly on the belt of the feeder beginning at approximately 0700 h. The amount of feed was adjusted after each of the live samplings but was not adjusted for mortality. Raceway fish were fed on a normal production schedule throughout the day based on fish size and raceway number using automated blower feeders.

### 2.4 Water Sampling for Pathogen Load Determination in Field Experiment

Triplicate water samples (1 mL) were collected daily for 29 days from the common intake for both the first and reuse water supplying field experiment tanks (**Figure 1A**). Individual effluent water samples were also collected every other day from each of the 20 outdoor experimental tanks. Water samples were initially stored on ice for up to 4 hours and subsequently frozen at -80°C until processing.

### 2.5 Live Fish Sampling for Pathogen Load Determination in Field Experiment

Tissue samples were collected from fish at 10 timepoints from field experiment tanks (days 1, 3, 6, 10, 12, 17, 19, 24, 26 and 27). Seven fish per tank were sampled at the first three sample time points, then 5 fish were sampled per tank at the remaining seven time points, unless there were no remaining fish (**Table 1**). Individual nets were used for each tank and shoulder-length gloves were changed between tanks to minimize the potential for cross-contamination. Fish were randomly netted from the tanks and placed in a labeled tank-specific Ziploc bag that contained 200mg/L of bicarbonate buffered MS-222. Once fish were euthanized, the remaining MS-222 solution was drained, and fish placed on ice and subsequently stored in a walk-in cooler at 4°C at Clear Springs’s research lab until processing. The nets and buckets were disinfected after sampling with 6% bleach for 10 min, rinsed, and dried.

External and internal tissues were separately collected from each of the live sampled fish. For the external sample, the second gill arch and a pectoral fin were removed with the same scalpel and forceps and placed in a single tube. The fish was then sprayed with a 70% ethanol solution and blotted with a KIMwipe. New disposable scalpel and forceps were used to expose the internal organs and the entire spleen and head kidney were removed and placed in a new 1.5 mL tube. The dissection workbench was decontaminated between tanks. Samples were kept on ice prior to being stored in a -80°C freezer.

After a total of 28 days in the outdoor tank system, the study was ended (on day 27). The final sampling included 5 fish that were euthanized and dissected as described above.

### 2.6 Sampling of Dead Fish for Pathogen Load Determination in Field Experiment

Dead fish were removed daily from each experimental tank. Up to 5 mortalities per tank were saved daily through day 5 and 10% were saved for processing after day 5. Mortalities were initially collected and placed on a Ziploc bag and stored on ice and subsequently stored in a -80°C freezer. The whole fish samples were shipped to VIMS where they were thawed and tissue collections (internal and external) were performed as described in section *2.5 Live fish sampling*.

### 2.7 qPCR for Fp and IHNV Load Measurement

Total RNA and DNA were extracted from 210 µl of water samples using the *cador* Pathogen 96 QIAcube HT Kit (Qiagen) on a Tecan EVO 100 liquid handler as previously described (31, 32). Tissue samples were first homogenized by adding 500µg of 1.0mm diameter Zirconia/Silica beads (Biospec) and 1000µl of Denaturing Solution (33), per 0.1 g of tissue, in a cryo-vial. The sample underwent 4.0 m/s bead beating for 20 seconds, two times. Afterwards, the sample was centrifuged at 2000rpm, for 3 minutes, at 20°C. A 200µl volume of the sample supernatant then underwent extraction using the *cador* Pathogen Mini Kit (Qiagen) following the manufacturer’s instructions, with a 100 µl elution volume using buffer AVE. Extracted samples were stored at -80°C until further processing. These chemistries co-extract DNA and RNA. This method was previously found to efficiently produce high purity nucleic acid (data not shown).

For IHNV load quantification, extracted RNA was combined with 0.125 μg random hexamers, and 0.125 μg oligo dT (Promega) to ensure complete conversion of RNA into cDNA, in a volume of 13 μL as previously described (33). The mix was incubated at 70°C for 5 min, held on ice and the volume brought to 20 μl with: 1 mM dNTPs, 4 μl 5× M-MLV reaction buffer, 100 U MMLV RT (Promega), 10 U RNAsin, and H2O (Promega). The sample was then incubated at 42°C for 1 h, 70°C for 15 min, and held at 4°C until storage at−80°C. The cDNA was thawed, diluted 1:2 with RNase/DNase-free water (Fisher Scientific), and a 5 µl volume underwent quantitative PCR (qPCR) using TaqMan probe IHNV N 818 MGB, forward primer IHNV N 796F, and reverse primer IHNV N 875R, targeting the viral nucleocapsid (N) gene, as previously described (34). All qPCR reactions included the APC viral N gene plasmid standard (34), to provide absolute quantification. The qPCR quantified the number of viral RNA copies, presented here as virus copies per milliliter of water or gram of tissue. The detection limit for this viral quantification protocol is approximately 400 viral RNA copies/ml of water and 3500 viral RNA copies/g tissue.

To quantify *Fp* load in water and tissue samples, DNA was extracted at VIMS as described above, frozen, and sent to the National Center for Cool and Cold Water Aquaculture, Kearneysville WV, USA. Samples were subjected to qPCR using FpSig probe as well as FpSig_fwd and FpSig_rev primers, which target a single copy gene in the *Fp* genome (35). DNA was amplified with TaqMan Universal Master Mix II + UNG (Applied Biosystems) and a standard curve of purified *F. psychrophilum* CSF 259-93 bacterial genomic DNA was used to convert cycle quantification (Cq) into log_10_ genome equivalents per mL of water (35). qPCR assays included a positive extraction control (25 Ct) as well as a standard curve of genomic DNA ranging from 3 genomic equivalents (GE) to 30 million GE per reaction well. The calculated *Fp* assay sensitivity for water samples was ~295 GE per mL and ~1000 GE per g tissue assuming 100% sample extraction efficiency.

### 2.8 Laboratory Challenge

To investigate fish line resistance to IHNV and *F. psychrophilum* in a more controlled setting, a laboratory experiment was conducted at VIMS under the guidelines of VIMS protocol # IACUC-2014-11-17-9966-arwargo. The CSF- and Tx-line fish from the same fish lot used in the field experiment were vaccinated using the same vaccine, dose, and procedure as described in section *2.1 Rainbow trout populations and vaccination* with the exception that a 30g needle was utilized. Sham-vaccinated fish were injected with PBS alone. Mean body weights at the time of vaccination, determined from an average of three, 10-fish pools, were 1.4 ± 0.0g and 1.5 ± 0.8g (mean ± SD) for the CSF and Tx fish, respectively. Post-vaccination, fish were fed a diet of #2 crumble (Zeigler) at 1.5% of body weight per day and held at 15°C in a flow through system supplied with UV-filtered specific pathogen-free well water. At thirty days post-vaccination (450 temperature degree days), fish were challenged with either *F. psychrophilum* strain CSF117-10 (36), IHNV isolate C (genotype mG119M, GenBank accession number: AF237984), both pathogens at the same time, or mock exposed. To prepare *F. psychrophilium* challenge inoculum, a single colony of isolate CSF117-10 was inoculated into 15 mL of TYES broth. After 3 days, 250 μL culture was transferred into 100 mL TYES in a 250 mL flask at 15°C, with 150 rpm shaking. After 4 days, O.D. 600nm was adjusted to 0.62 in TYES media and fish were injected with 50 μL of the inoculum by intraperitoneal injection with a 27 g needle fitted onto an Eppendorf syringe. The bacterial titer of the inoculum was subsequently determined to be 1.16 x 10^6^ CFU/fish, through serial dilution on TYES-AGAR plates. Control fish (and single IHNV infection fish) were injected with 50 μL TYES. For IHNV challenges, the virus was propagated on *Epithelioma Papulosum Cyprini* (EPC) cells (37) titered with triplicate plaque assays and stored at -80°C, as previously described (38). Fish were exposed to 2 x 10^5^ pfu/ml diluted in 5ml minimum essential media + 10% fetal bovine serum (MEM10), by placing them in 1L of water with virus for 1 hour, under static conditions. Water flow (100 ml/min) was then resumed to each tank as previously described (39). Control fish (and single infection *F. psychrophilum* fish) were exposed to 5ml MEM10. Mixed infection fish were first injected with *F. psychrophilum* (or TYES), then 5 minutes later exposed to IHNV (or MEM10), at the same dosages as single infection fish.

Prior to vaccination or challenge, fish were taken off feed for 24h, and anesthetized with 100 mg MS-222 with 300 mg sodium bicarbonate per 1L of water. Mean body weights at the time of challenge of CSF sham and CSF vaccinated fish, were 1.8 ± 0.0g and 1.8 ± 0.2g, respectively and determined from a mean ± SD of three 10-fish pools. Mean body weights of Tx sham and Tx vaccinated fish were 2.2 ± 0.2g and 2.3 ± 0.2g, respectively. Triplicate tanks were used per treatment, and fish were randomly assigned to tank by genetic line and vaccination status with n=13-14 fish per tank.

### 2.9 Statistical Analyses of Field Experiment and Laboratory Challenge Datasets

Analyses of the survival differences in the field experiment and laboratory challenge were conducted using semiparametric Cox regression models implemented in PROC PHREG (SAS Version 9.4; SAS Institute, Inc., Cary, NC) using the *param*=*GLM* and *ties*=*exact* options. Fish that were live sampled from the field experiment were treated as censored on the day of collection. All fish that survived for the duration of the 27-day field experiment and 34-day laboratory challenge were treated as censored at the end of the respective study. No mortalities were observed in four treatment subclasses of the field experiment (CSF × Control × Shm; Tx × Control × Shm; CSF × Control × Vac; CSF × Pulsed × Vac), nor in the CSF × Vac × IHNV subclass of the laboratory challenge. To allow parameter estimation, a single mortality record at day 1 was added to the datasets for each of these subclasses.

Field experiment survival data were analyzed using a model that included fixed effects of water exposure treatment (4 levels after merging data from the constant and donor treatments), genetic line (2 levels), vaccine treatment (2 levels), and all 2- and 3-way interactions to estimate Wald χ^2^ and *P*-values for Type III tests of main effects. Subsequently, a single independent variable with 16 levels (i.e., 1 level for each main effect subclass) was created and orthogonal contrasts were constructed to estimate hazard ratios between levels of factors and their interactions that were significant (*P* < 0.05) in the initial model.

Laboratory challenge survival data were analyzed using a model that included fixed effects of pathogen exposure (3 levels), genetic line (2 levels), vaccine treatment (2 levels), all 2- and 3-way interactions, and a random effect challenge tank (3 levels) nested within each of the 12 pathogen exposure × genetic line × vaccine treatment subclasses. The random challenge tank effect was not significant (*P* = 0.09) and thus was removed from the model to estimate Wald χ^2^ and *P*-values for Type III tests of main effects. Subsequently, a single independent variable with 12 levels (i.e., 1 level for each main effect subclass) was created and orthogonal contrasts were constructed to estimate hazard ratios between levels of factors and their interactions that were significant (*P* < 0.05) in the fixed effects-only model. For both field and laboratory experiment survival data, only comparisons between biologically relevant factor levels were examined.

Analysis of mean time-to-death was calculated using GraphPad v7.05 software and pair-wise comparison determined using a Mann-Whitney test. Pathogen loads were *log_10_* transformed and analyzed using one-way ANOVA with planned contrasts between external *vs* internal loads and between sham and vaccinated fish (GraphPad v 7.05).

## 3 Results

### 3.1 Reuse Water Environmental Parameters and Pathogen Load

To assess reuse water quality, the water was sampled prior to entering experimental tanks, and temperature and dissolved oxygen monitored daily throughout the study period (**Figures 1C, D** **)**. The mean temperature did not differ between first use and reuse water, 14.3 ± 0.2 *vs* 14.4 ± 0.2°C (mean ± SD), respectively (**Figure 1C**). The reuse water exhibited lower mean dissolved oxygen saturation compared to first-use water throughout the study period, 57.7 ± 5.8% (5.9 ± 0.6 mg/L) and 89.3 ± 2.2% (9.1 ± 0.2 mg/L) respectively (**Figure 1D**).

Mortality in the production raceways supplying the reuse water was recorded daily, and within raceway series designated 3A-C, elevated mortality was observed above baseline (**Supplementary Figure 1**). Prior to the start of this experiment, fish from raceway 3A were diagnosed with a polymicrobial mixed infection consisting of IHNV, bacterial gill disease, columnaris, and external bacterial infection. At the start of the field study, mortality in raceway 3C peaked at 180 fish per day, and in raceway 3B, elevated morality occurred through study day 8 with a total of 4.5% cumulative mortality during the field experiment period.

We attempted to quantify IHNV and *F. psychrophilum* load in triplicate samples of first use and reuse water supplied to the experimental tank system, with pathogen specific qPCR assays. All first use water samples, obtained from a 6-day period that included day -1 through day 4 (n=16 water samples), were below detection for both pathogens. Analysis of the reuse water samples matched in time and number (n=16) were also below detection for *F. psychrophilum* and IHNV except for one sample that was weakly positive for *F. psychrophilum* (475 GE per mL). Taken together, the levels of *F. psychrophilum* and IHNV were at or below detection in both first use and reuse water, and as such additional days of intake water samples were not processed. However, given the elevated mortality in the commercial raceways, the reuse water likely contained pathogen(s) not detected by our qPCR assays.

### 3.2 Effect of Reuse Water Exposure, Genetic Line, and Vaccine Treatment on Survival in Field Experiment

**Figure 2:**
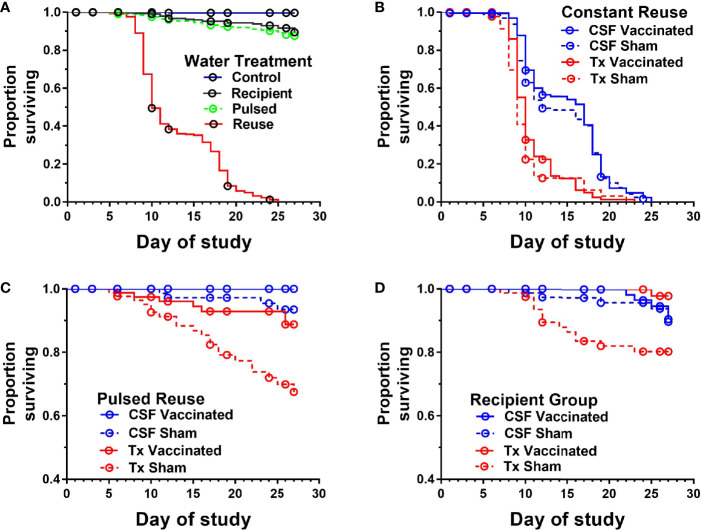
Survival of two genetic lines of rainbow trout exposed to various water reuse paradigms and IHNV vaccination. Plots show proportion of fish surviving different water treatments. Overall comparison of water treatments in which genetic line and vaccine treatments were collapsed **(A)**, Constant reuse water exposed group comparing genetic line and treatment **(B)**. Note the Constant and Donor groups were combined by genetic line and vaccine status. Pulsed reuse water exposed group comparing genetic line and vaccine treatment **(C)**, and Recipient group comparing genetic line and vaccine treatment **(D)**.

The survival of vaccinated or sham-vaccinated CSF- and Tx-line fish were monitored daily for 27 days under five different exposure treatments (**Table 1**). Fish in the Control group (spring water) exhibited >99% survival (**Figure 2A**), while in contrast, all fish exposed to constant reuse water (Constant and Donor groups) died within the study period (**Table 1** and **Figure 2B**). In the statistical model, reuse water exposure, genetic line, vaccination, and the interaction between water exposure and genetic line affected survival (*P*< 0.05, **Table 2**). Water usage treatments was nuanced because the effect depended on fish line (*P* = 0.042, **Table 2**, line*water interaction) but some patterns did emerge. CSF and Tx fish exposure to constant reuse water resulted in a significant, 45.9 to 709.7-fold, increase in death compared to all other water usage types (**Table 3**, line*water interaction). Pulsed reuse water resulted in 14.9-fold increased hazard of death, compared to control water exposure for the Tx fish line (*P*=0.0003) but not for the CSF line (*P*=0.44; **Table 3** and **Figure 2C**). The opposite was true for recipient water usage compared to control first use water, where the CSF line had increased hazard of death (*P*=0.0272), but the Tx line did not reach significance (*P*=0.097; **Table 3** and **Figure 2D**). Overall, vaccination resulted in a 2-fold decrease in the hazard of death, across all water usage types and fish genetic lines (*P* = 0.0218, **Tables 2** and **3**). There was some indication that vaccine efficacy differed across water usage types, likely because few fish died in control water treatment, but this was not significant (*P* = 0.052, **Table 2**, water*vaccination interaction). The Tx fish line had a 2.7 to 7.2-fold greater hazard of death compared to the CSF line in constant (**Figure 2B**) and pulsed reuse water (**Figure 2C**) respectively, while there was little difference between lines in the control and recipient water treatments, where fish mortality was minimal (**Table 3**, Control: CSF *vs* Tx and Recip: CSF *vs* Tx).

**Table 2:** Significant effects survival analysis in the field experiment.

Effect	DF^a^	Wald Chi-Square	Pr>ChiSq^b^
Water Reuse	3	447.7393	**<.0001**
Genetic Line	1	4.2012	**0.0404**
Vaccination	1	5.2601	**0.0218**
Line*Water	3	8.2047	**0.042**
Water*Vaccination	3	7.7262	0.052
Line*Vaccination	1	0.8484	0.357
Line*Water*Vaccination	3	3.7954	0.2844

a DF, degrees of freedom.

b Pr>ChiSq, probability that the Chi-Square test statistic is greater than or equal to what has been observed under the null hypothesis.

*indicates interaction between or among effects. Bold values P < 0.05.

**Table 3:** Model contrasts, hazard ratio and P-value for survival analysis comparisons from field experiment.

Factor	Contrast	Hazard Ratio	StdErr	lower 95% CI	upper 95% CI	WaldChiSq	DF^a^	Pr>ChiSq^b^
Water	Constant *vs.* Control	422.400	216.200	154.900	1152.100	139.518	1	**<.0001**
	Constant *vs.* Pulse	77.286	24.569	41.448	144.100	187.021	1	**<.0001**
	Constant *vs.* Recip	87.219	27.746	46.755	162.700	197.301	1	**<.0001**
	Pulse *vs.* Control	5.466	3.188	1.743	17.141	8.484	1	**0.0036**
	Recip *vs.* Control	4.844	2.824	1.545	15.185	7.323	1	**0.0068**
	Pulse *vs.* Recip	1.129	0.479	0.491	2.592	0.081	1	0.7756
Line	Tx *vs.* CSF	1.962	0.645	1.030	3.736	4.201	1	**0.0404**
Vaccination	Sham *vs.* Vacc	2.125	0.698	1.116	4.045	5.260	1	**0.0218**
Line*Water	CSF: Constant *vs.* Control	251.500	180.000	61.820	1022.900	59.614	1	**<.0001**
	CSF: Constant *vs.* Pulse	125.400	71.450	41.029	383.100	71.866	1	**<.0001**
	CSF: Constant *vs.* Recip	45.980	14.811	24.456	86.448	141.241	1	**<.0001**
	CSF: Recip *vs.* Control	5.469	4.207	1.211	24.698	4.879	1	**0.0272**
	CSF: Pulse *vs.* Control	2.006	1.808	0.343	11.737	0.596	1	0.4400
	CSF: Pulse *vs.* Recip	0.367	0.233	0.106	1.275	2.490	1	0.1146
	Tx: Constant *vs.* Control	709.700	509.800	173.600	2900.800	83.517	1	**<.0001**
	Tx: Constant *vs.* Pulse	47.643	11.850	29.261	77.572	241.330	1	**<.0001**
	Tx: Constant *vs.* Recip	165.400	87.989	58.337	469.200	92.267	1	**<.0001**
	Tx: Recip *vs.* Control	4.290	3.759	0.770	23.895	2.761	1	0.0966
	Tx: Pulse *vs.* Control	14.896	11.024	3.492	63.535	13.320	1	**0.0003**
	Tx: Pulse *vs.* Recip	3.473	1.951	1.155	10.442	4.912	1	**0.0267**
	Control: CSF *vs.* Tx	1.026	1.026	0.145	7.287	0.001	1	0.9792
	Constant: Tx *vs.* CSF	2.749	0.261	2.283	3.311	113.803	1	**<.0001**
	Pulse: Tx *vs.* CSF	7.235	4.342	2.231	23.459	10.872	1	**0.001**
	Recip: CSF *vs.* Tx	1.309	0.785	0.404	4.239	0.201	1	0.6536

a DF, degrees of freedom.

b Pr>ChiSq, probability that the Chi-Square test statistic is greater than or equal to what has been observed under the null hypothesis.

*indicates interaction between factors. Bold values P < 0.05.

The mean days-to-death were compared between genetic lines in constant reuse water after excluding censored animals. CSF-line fish (pooled vaccine and sham data) exhibited a 4.1-day increase in median survival compared to the Tx-line (13.8 ± 4.8 days *vs* 9.7 ± 2.3; *P*<0.001, Mann-Whitney test, mean ± SD). The Tx-line fish died between days 7 and 11 in a single phase of mortality while the CSF line (both vaccine and sham) was more heterogeneous and exhibited two phases of mortality, the first occurring between days 8-12, and the second occurring between days 16-20 (**Figure 2B**). The Tx vaccinated fish exhibited a 1-day increase in median survival as compared to the Tx sham-vaccinated group (10.2 ± 2.5 *vs* 9.2 ± 2.1 days; *P*<0.001) while the mean day-to-death did not significantly differ between CSF vaccinated and CSF sham-vaccinated groups (14.1 ± 4.7 *vs* 13.5 ± 4.8, *P*=0.19).

In summary, exposure to constant reuse water decreased survival, vaccination in general increased survival, and the CSF line had better survival than the Tx line.

### 3.3 Clinical Disease Signs

The fish exposed to constant reuse water exhibited decreased appetite after 5-6 days. Clinical disease signs were observed by days 9-10 that included extended fecal casts, exophthalmia and associated hemorrhaging, redness/hemorrhaging at the site of pectoral fins attachment, and lesions under the mouth. Fish in constant reuse water also had a reduced mucus layer with abrasions and loss of ability to color-match tank shading, as indicated by marbled coloring across sides and dorsal surface (**Supplementary Figure 2A**) but the precise frequency of these external clinical signs were not quantified. Internal clinical disease signs included enlarged spleens (**Supplementary Figure 2B**) which is a general sign of inflammation and observed after *F. psychrophilum* challenge (40). In summary, these disease signs are consistent with both environmental stresses (41), IHNV (42) and BCWD (40). None of these clinical signs were observed in control fish that were held in spring water.

### 3.4 Pathogen Load in External *Versus* Internal Tissues

**Figure 3:**
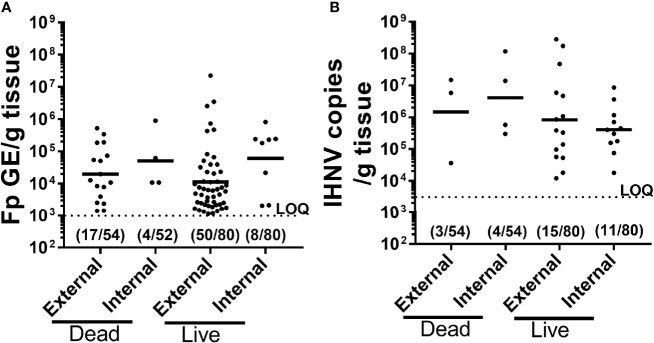
Comparison of pathogen prevalence and load from live and dead-sampled fish held in constant reuse water. Plots compare *F*. *psychrophilum* **(A)** and IHNV **(B)** prevalence and load **(A)**; Log10(bacterial DNA copies/g tissue); **(B)** Log10 [viral RNA copies/g tissue or DNA copies)] for mortalities (died between days 8-19) and live-sampled fish (days 10 and 12) reared under constant reuse water (Donor and Constant groups combined). External pathogen was measured in gill/fin-clip homogenates while internal pathogen load was measured in kidney/spleen homogenates. Individual fish loads are indicated in addition to geometric mean. Limit of quantification for both qPCR assays is indicated by dotted line. Numbers indicate positive fish and total sampled for each group. The full datasets for live-sampled and dead-sampled fish are in **Supplemental Tables 1** and **2**, respectively.

To better understand causes of mortality, we analyzed *F. psychrophilum* and IHNV prevalence and load by qPCR in both live-sampled fish (**Supplementary Table 1**) and a subset of fish that died and then removed from tanks for sampling (**Supplementary Table 2** **).** We focused on the constant reuse water exposed groups (Constant and Donor) (**Table 4**), and days 8-19, because that is where most mortality occurred (**Figure 2B**). Of all the combined live-sampled fish processed, a total of 62% (50/80) had quantifiable external loads of *F. psychrophilum* while 10% (8/80) had quantifiable internal loads (**Table 4** and **Supplementary Tables 1** and **2**). Of the combined dead-sampled fish, a total of 31.0% (17/54) had quantifiable external loads of *F. psychrophilum* while 8% (4/52) had quantifiable internal loads. In these same fish that were assayed for IHNV, a total of 19% (15/80) live sampled fish had quantifiable loads of IHNV, while 14% (11/80) had internal loads. Of the mortalities sampled, 6% (3/54) had quantifiable external IHNV while 8% (4/54) exhibited internal loads (**Table 4**). Among fish that had quantifiable loads, the amount of *F. psychrophilum* did not significantly differ between internal *vs* external or live *vs* dead sampled fish (one-way ANOVA, *F*_3,75 _= 1.86 *P*=0.14, **Figure 3A**). This was also true for IHNV (one-way ANOVA *F*_3,29 _= 0.74 *p*=0.53, **Figure 3B**), but mean IHNV loads were generally about one order of magnitude higher per g tissue as compared to *F. psychrophilum* (**Figure 3B** *vs* **Figure 3A**). The prevalence of detectable IHNV was also generally lower than *F. psychrophilum* except for live-sampled Tx sham-vaccinated group in which 60% of the fish were positive for external IHNV and 45% positive for internal IHNV (**Table 4**).

**Table 4:** Frequency of fish positive for *F. psychrophilum*, IHNV, or co-infected with both pathogens, that had been removed from tanks alive, euthanized and sampled, or sampled after death in constant reuse water.

Sampled	Line	Vac	Sampling(days)	*Fp* ^+^		IHNV^+^		Co-infected (Ext or Int) % (n)
				External	Internal	External	Internal	
				% (n)	% (n)	% (n)	% (n)	
Live	CSF	Shm	10,12	60% (12/20)	5% (1/20)	10% (2/20)	10% (2/20)	10% (2/20)
		Vac	10,12	80% (16/20)	5% (1/20)	5% (1/20)	0% (0/20)	0% (0/20)
	Tx	Shm	10,12	55% (11/20)	25% (5/20)	60% (12/20)	45% (9/20)	40% (8/20)
		Vac	10,12	55% (11/20)	5% (1/20)	0% (0/20)	0% (0/20)	0% (0/20)
Live Total				62% (50/80)	10% (8/80)	19% (15/80)	14%(11/80)	10% (10/80)
Dead	CSF	Shm	8,9,18,19	27% (4/15)	14% (2/14)	7% (1/15)	7% (1/15)	7% (1/15)
		Vac	8,9,18,19	20% (3/15)	7% (1/15)	0% (0/15)	0% (0/15)	0% (0/15)
	Tx	Shm	8,9,10	36% (4/11)	10% (1/10)	18% (2/11)	20% (2/10)	18% (2/11)
		Vac	8,9,10,16	46% (6/13)	0% (0/13)	0% (0/13)	8% (1/13)	8% (1/13)
Dead Total				31% (17/54)	8% (4/52)	6% (3/54)	8% (4/53)	7% (4/54)

Data from the Constant and Donor groups were combined.

To summarize, a high percentage of fish in constant reuse water had external *F. psychrophilum* but the bacterial loads were low while 10% had quantifiable internal loads. IHNV was infrequently detected but the viral loads were moderately high. Diagnosis of fish was highly consistent with IHNV and *F. psychrophilum* infection and neither *F. psychrophilum* nor IHNV were detected in fish exposed to only spring water (Control – 8 fish tested). These findings were supported by live fish samples from the recipient (51 fish tested) and pulsed reuse water (16 fish tested) treatments, where IHNV and *F. psychrophilum* prevalence ranged from 17-50% (**Supplementary Table 1**). However, other causes of death could not be ruled out as neither pathogen was detected in 59% (32/54) of the fish that died in reuse water.

### 3.5 Effect of DNA Vaccination and Genetic Line on Pathogen Load

We next examined whether there was evidence for a vaccine effect on pathogen prevalence and load under constant reuse water exposure. In live-sampled Tx-line fish, vaccination reduced the prevalence of IHNV from 60% to 0% in external samples and from 45% to 0% in internal samples (**Table 4**). A similar trend was observed in the CSF line where vaccination reduced prevalence from 10% to 5% in external tissues and 10% to 0% in internal tissues. In sham fish, IHNV prevalence was higher in the Tx compared to CSF fish line (**Table 4**). This was not true in vaccinated fish because IHNV was completely cleared. The effect of IHNV vaccination on the prevalence of *F. psychrophilum* infection was more nuanced (**Table 4**). There was little evidence of an effect of vaccination on *F. psychrophilum* prevalence in live sampled fish, except for internal tissues in the Tx-line where vaccination reduced prevalence from 25% to 5%. In dead fish, there was a general trend that vaccination reduced *F. psychrophilum* prevalence in both the CSF and Tx lines, as well as external and internal samples. However, it should be noted that *F. psychrophilum* prevalence in dead fish was generally low and prevalence differences between sham and vaccinated groups were typically due to only 1-2 fish. Due to these reductions in IHNV and *F. psychrophilum* prevalence, vaccination also reduced co-infection in all fish lines (**Table 4**).

**Figure 4:**
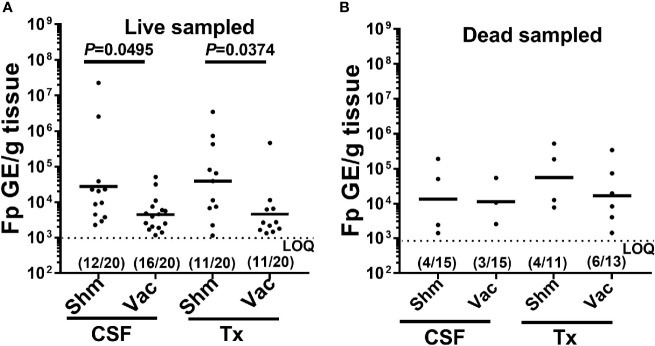
Effect of IHNV vaccination on external *F. psychrophilum* load. Plots compare *F. psychrophilum* prevalence and load (Log10 bacterial DNA copies/g tissue) in gill/fin samples from vaccinated and sham live-sampled fish **(A)**: days 10 and 12) and mortalities **(B)**: died between days 8-19) and reared under constant reuse water (Donor and Constant groups combined). Geometric mean indicated by bar and the limit of the *F. psychrophilum* qPCR assay indicated by dotted line. P-values denote significant differences between groups. Numbers indicate fish positive and total sampled for each group.

When examining external pathogen load quantification, for only those fish which were pathogen positive, vaccination significantly reduced external *F. psychrophilum* loads in both the CSF and Tx-lines, in live sampled fish (**Figure 4A**, one-way ANOVA *F*_3,46 _= 3.87, *P*=0.015). There was no effect of vaccination on *F. psychrophilum* loads in dead fish (**Figure 4B**, one-way ANOVA *F*_3,13 _= 0.479, *P*=0.703). In general, there was also no difference in mean *F. psychrophilum* loads between CSF and Tx-fish. Because vaccination reduced IHNV prevalence to zero, mean IHNV load could not be compared. Pathogen loads were also not compared in internal tissues, because very few vaccinated fish were positive for either pathogen. Taken together, these data indicate that under constant reuse water conditions, IHNV vaccination reduced IHNV prevalence, and interestingly, there was reduction of external *F. psychrophilum* load, but not prevalence, in live sampled Tx- and CSF-line fish.

### 3.6 Infection Dynamics and Natural Co-Infection

**Figure 5:**
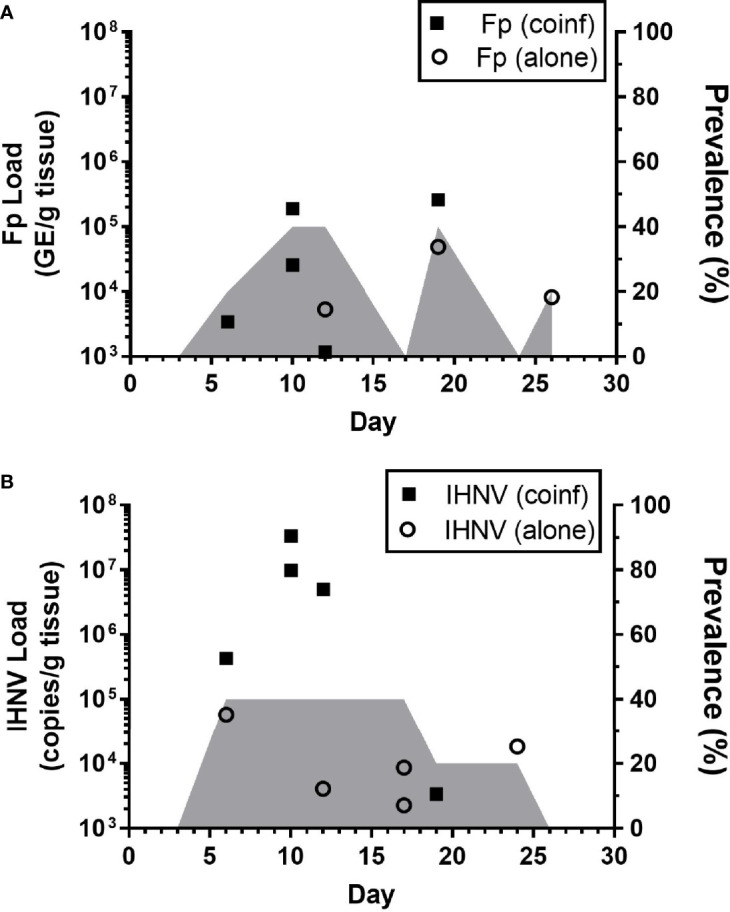
Kinetics of internal pathogen load in field studies. Plots show IHNV **(A)** and *F. psychrophilum* **(B)** load [**A**; Log10 (bacterial DNA copies/g tissue); **B**: Log10 (viral RNA copies/g tissue or DNA copies)] as well as pathogen prevalence (2nd y-axis, shaded area) in live-sampled, sham-vaccinated Tx-line recipient treatment group fish. Individual co-infected fish are indicated by solid symbols while singly infected fish are indicated by open symbols. N=5 fish per time point. Fish with no pathogen detected are not shown.

The kinetics of infection were examined in detail within the Recipient group of the sham-vaccinated, Tx-line in the field experiments (**Figure 2D**, red dotted line). This treatment group was chosen for the kinetic analyses because the exact timing of exposure of these fish to pathogens could be estimated as time that the donor fish were added to the tank. The 80.4% survival proportion in this treatment also indicated that infection of these fish occurred. *Flavobacterium psychrophilum* (**Figure 5A**) and IHNV (**Figure 5B**) loads were quantified from internal tissues of live-sampled fish. *Flavobacterium psychrophilum* and IHNV were first detected by day 6 with peak loads occurring by day 10 and coinciding with the onset of mortality (**Figure 2D**). The highest prevalence, 40%, was detected on days 10,12 and 18 for *F. psychrophilum* (**Figure 5A**, shaded area) and days 6-17 for IHNV (**Figure 5B**). Load and prevalence subsequently decreased by day 26. Eleven percent (n=5/45) of tested fish were coinfected with both pathogens and these fish tended to have the highest IHNV loads (**Figure 5B**, solid squares) as compared to fish infected with IHNV alone (open circles). This pattern of coinfection exacerbating pathogen loads was not apparent for *F. psychrophilum*. Taken together, these data indicate that internal infections were initiated by 6 days after donor fish exposed to reuse water were added to recipient tanks, with maximal pathogen loads occurring at days ~10-12 and evidence of internal co-infection. Furthermore, because peak estimated prevalence of *F. psychrophilum* and IHNV infection was greater than 20% (percentage of donor fish added to the tank), this would imply that there was transmission of pathogen between donor and recipient fish in the tank, given that no fish in the control tanks exposed to single use water were found infected.

### 3.7 Shedding in Water Samples From Tank Effluents

We attempted to measure pathogen shedding from infected fish in water samples taken from effluent water of each tank from the field study on days -1, 1 and 3. All samples (n=60) were below detection for *F. psychrophilum*. IHNV was detected in 3.3% of water samples from first use tanks (n=5/149) and in 1% of samples from reuse water (n=1/96), with no pattern between treatment groups. Despite this very low prevalence in effluent water, this does indicate some pathogen amplification in experimental tanks given that all influent water tested negative for IHNV (section 3.1).

### 3.8 Laboratory Challenge of Genetic Lines With Single and Multiple Pathogens

**Figure 6:**
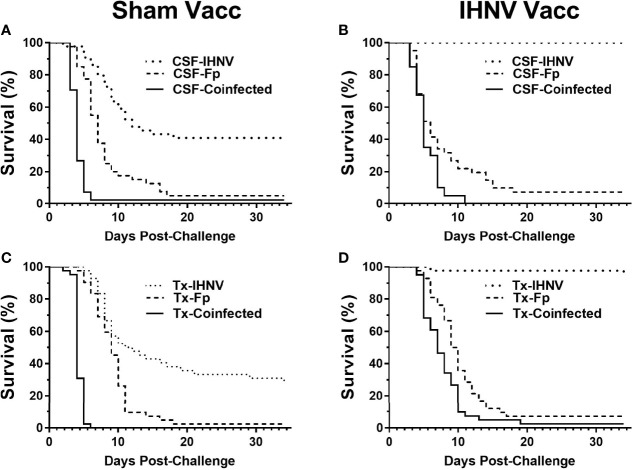
Fish survival laboratory studies. Plots show survival of two rainbow trout genetic lines, CSF **(A, B)** and Tx **(C, D)** that had either been sham vaccinated **(A, C)** or DNA vaccinated against IHNV **(B, D)**. Fish were challenged with IHNV (dotted line), *F. psychrophilum* (stippled line) or both pathogens at a 1:1 ratio (solid line).

The resistance of the CSF- and Tx-line fish and their respective response to IHNV DNA vaccination were compared under defined laboratory conditions using fish from the same egg lots used in the field study. The fish were challenged with IHNV alone, *F. psychrophilum* alone, or with both pathogens simultaneously. Challenge with both pathogens resulted in more rapid and nearly complete mortality independent of genetic line and vaccination status in the laboratory challenge (**Figure 6** and **Supplementary Figure 3**). In the statistical model, the hazard of death was complex as it depended on the interaction between fish line, vaccination, and pathogen exposure (*P*=0.037, **Table 5**), and thus, twenty contrasts are listed in **Table 6**. In the absence of vaccination, there was no significant difference in the hazard of death between the CSF or Tx fish lines, for any of the pathogen treatments (**Figures 6A** *vs.* **C**, **Table 6**). This was also true in the presence of vaccination (**Figures 6B** *vs.* **D**, **Table 6**), except for fish exposed simultaneously to both pathogens where the hazard of death was 2-fold lower for the Tx line. Furthermore, in the absence of vaccination, co-infection with both pathogens increased the hazard of death by 5 to 20-fold compared to single infections of either pathogen, in both fish lines (**Figures 6A, C** and **Table 6**). As such, co-infection universally exacerbated disease caused by IHNV and *F. psychrophilum* infection. Vaccination reduced the hazard of death of fish exposed to IHNV alone by 23 to 42-fold across both fish lines, virtually eliminating all mortality (**Figures 6A** *vs.* **B** and **C** *vs.* **D**, **Table 6**). Vaccination also reduced the hazard of death of fish co-exposed to both pathogens, but only by 2 to 7-fold (**Figures 6A** *vs.* **B** and **C** *vs.* **D**, **Table 6**). As such, co-infection reduced IHNV vaccine efficacy. There was no effect of vaccination on the survival of fish exposed to *F. psychrophilum* alone, in either fish line (**Figures 6A** *vs.* **B** and **C** *vs.* **D** and **Table 6**). The hazard of death was 2 to 3-fold higher for fish exposed to *F. psychrophilum* alone compared to IHNV alone, in the absence of vaccination (**Figures 6A, C** and **Table 6**). Based on hazard ratios, there was no consistent pattern that one fish line responded better to vaccination than the other (**Table 6**).

**Table 5:** Significant effects survival analysis in the laboratory experiment.

Effect	DF^a^	Wald Chi-Square	Pr>ChiSq^b^
Genetic Line	1	0.0639	0.8005
Vaccination	1	52.093	**<.0001**
Line*Vaccination	1	0.3666	0.5448
Pathogen	2	208.0453	**<.0001**
Line*Pathogen	2	1.5114	0.4697
Vaccination*Pathogen	2	47.4268	**<.0001**
Line*Vaccination*Pathogen	2	6.5993	**0.0369**
Tank^c^ (random)	8.5	14.4417	0.0879

a DF, degrees of freedom.

b Pr>ChiSq, probability that the Chi-Square test statistic is greater than or equal to what has been observed under the null hypothesis.

c Tank effect was removed from final model (NS, and was not a design variable).

*indicates interaction between or among effects.

**Table 6:** Model contrasts, hazard ratio and P-value for comparisons from laboratory experiment.

Factor	Contrast	Hazard Ratio	StdErr	lower 95% CI	upper 95% CI	WaldChiSq	DF^a^	Pr>ChiSq^b^
Line*Vacc*Path								
	Fp: CSF sham *vs.* Tx sham	1.325	0.300	0.850	2.065	1.541	1	0.2145
	Fp: CSF vacc *vs.* Tx vacc	1.523	0.349	0.972	2.385	3.370	1	0.0664
	IHNV: Tx sham *vs.* CSF sham	1.154	0.309	0.682	1.952	0.286	1	0.593
	IHNV: Tx vacc *vs.* CSF vacc	2.041	2.499	0.185	22.495	0.339	1	0.5602
	Both Path: Tx sham *vs.* CSF sham	1.453	0.343	0.915	2.309	2.503	1	0.1137
	Both Path: CSF vacc *vs.* Tx vacc	2.378	0.541	1.522	3.714	14.485	1	**0.0001**
	CSF: both sham *vs.* Fp sham	5.009	1.172	3.167	7.923	47.446	1	**<.0001**
	CSF: both sham *vs.* IHNV sham	16.109	4.204	9.658	26.867	113.406	1	**<.0001**
	CSF: Fp sham *vs.* IHNV sham	3.216	0.825	1.945	5.318	20.713	1	**<.0001**
	Tx: both sham *vs.* Fp sham	9.644	2.403	5.918	15.715	82.740	1	**<.0001**
	Tx: both sham *vs.* IHNV sham	20.285	5.484	11.941	34.459	123.941	1	**<.0001**
	Tx: Fp sham *vs.* IHNV sham	2.103	0.512	1.306	3.388	9.349	1	**0.0022**
	CSF: Fp vacc *vs.* Fp sham	1.001	0.231	0.637	1.572	0.000	1	0.9963
	CSF: IHNV sham *vs.* IHNV vacc	41.832	42.628	5.677	308.300	13.424	1	**0.0002**
	CSF: both sham *vs.* both vacc	2.008	0.464	1.277	3.158	9.111	1	**0.0025**
	Tx: Fp sham *vs.* Fp vacc	1.148	0.258	0.740	1.782	0.379	1	0.5382
	Tx: IHNV sham *vs.* IHNV vacc	23.656	17.287	5.649	99.072	18.744	1	**<.0001**
	Tx: both sham *vs.* both vacc	6.938	1.714	4.275	11.258	61.500	1	**<.0001**
	CSF: both vacc *vs.* IHNV vacc	335.600	340.900	45.821	2457.900	32.773	1	**<.0001**
	Tx: both vacc *vs.* IHNV vacc	69.170	50.311	16.626	287.800	33.926	1	**<.0001**

a DF, degrees of freedom.

b Pr>ChiSq, probability that the Chi-Square test statistic is greater than or equal to what has been observed under the null hypothesis.

*indicates interaction among factors. Bold values P < 0.05.

## 4 Discussion

Infectious disease outbreaks in production aquaculture systems are complex and the environmental factors that influence host-pathogen interactions are poorly understood. Reduced water quality is often blamed for disease outbreaks and is influenced by fish density. High rainbow trout density is also associated with reduced food intake, feed conversion, and poor fin condition (43). Here we utilize a replicated tank system (28) to expose fish, held at a relatively low density of 8.8 kg/m^3^, to reuse water derived from production fish raceways under standard conditions. This design made it possible to simulate the production environment (flow rate, temperature, DO, water source, etc.) in our experimental tank system, although it should be noted that fish density in production raceways is typically higher than our experimental tanks, which was the case in this study. We compared exposure of constant spring water (first use) to the effects of pulsed or constant reuse water (fourth use). The primary finding of this study was water reuse exposure, genetic line, vaccination, and the interaction between genetic line and water exposure affected survival. Importantly, genetic line and vaccination effects were completely overwhelmed by constant exposure to reuse water. This was likely driven by co-infection of fish with multiple pathogens, as well as other environmental factors associated with reuse water. The deleterious impact of co-infection was further supported by controlled laboratory challenges with the two primary pathogens found in rainbow trout aquaculture, IHNV and *Flavobacterium psychrophilum.*

### 4.1 Differences Between Spring Water and Reuse Water

A major environmental difference between spring water and reuse water is the amount of dissolved oxygen. In this study, the dissolved oxygen saturation of reuse water was 5.9 ± 0.6 mg/L (mean ± SD) which is 30 percentage points lower than the spring use water (9.1 ± 0.2 mg/L), and this was consistent over the study period. This level is above the lethal limit of 2-3 mg/L for rainbow trout (43) but approaches the 4 to 4.5 mg/L level considered to have detrimental effect on food intake, growth, and food conversion efficiency (44). Reuse water is also known to have higher total dissolved solids, turbidity, and lower pH (28), but these parameters were not measured in this study. Compared to spring water, the reuse water likely contained higher number of pathogenic microorganisms as the mortality in raceway series 3C feeding the experimental system peaked at the start of the field study at 180 fish per day, and raceway 3B exhibited a total of 4.5% cumulative mortality over the study period. This level of raceway mortality is considered relatively “low” at the commercial site and highlights the variation that occurs between raceways at the farm site (series 3A-C compared to 4A-C). The raceway mortality was attributed to “mixed infections” that are commonly observed during rainbow trout grow-out. Efforts to quantify the pathogen load in the intake water were not successful as we were unable to detect IHNV and only low-level *F. psychrophilum* in one water sample. The large water volumes and flow rates likely hindered the ability to detect pathogens present. Nevertheless, pathogens must have been transferred through the reuse water as we did not detect IHNV or *F. psychrophilum* and no mortality was observed in the fish held exclusively in spring water but did occur in those exposed to reuse water. Likewise, the clinical signs of dead fish were consistent with pathogen infection and both IHNV and *F. psychrophilum* were detected in many of the mortalities as well as the effluent of some experimental tanks. The kinetics of mortality, particularly in the recipient treatment were also aligned with IHNV and *F. psychrophilum* infection.

### 4.2 Host Genetics and Reuse Water

Selective breeding of fish for disease resistance is an increasingly utilized strategy to reduce risk of specific diseases in aquaculture (45, 46) but the interaction of host genetics with environmental parameters is poorly understood. The survival or time-to-death of the CSF line was generally superior to the Tx-line in both pulsed and constant reuse water in the field experiment. The CSF line has been subjected to multiple generations of selection for IHNV resistance and presumably the local farm environment, while the Tx-line, to our knowledge, has not been selected for IHNV resistance. This was in line with our finding that IHNV prevalence was in general higher for the Tx-line in the field compared to the CSF-line. We also expected a higher survival of the CSF-line (sham vaccinated) in the laboratory IHNV challenge, but this was not observed. We consider the laboratory experiment to have sufficient power to detect a difference, given that 42 fish per treatment provided a power of 0.65 to distinguish an expected 24-percentage point difference in survival proportion between genetic lines at an alpha value of 0.05 (GPower v3.1). This assumption was based on an expected 3 percentage point improvement per generation multiplied by 8 generations of selection reported previously (23). So, we do not suspect lack of statistical power was the reason differences were not observed. We suggest some other potential explanations. First it is possible that the pathogen strain used in the lab challenge differed from the strains present in the reuse water in the field (39, 47, 48). It is possible that a component of fish genetic resistance may be pathogen strain specific (49). Secondly, the CSF and Tx-lines at the farm site were maintained on a standard commercial feed schedule, while in the laboratory, the CSF and Tx-lines were maintained on a restricted feed schedule and challenged at a different size. It has been shown previously that age and size impact IHNV and BCWD resistance in rainbow trout (24, 50). There are also a variety of other environmental stressors in the field which we did not attempt to simulate in the lab, which may interact with pathogen virulence. Our results do suggest, as others have (5, 39), that stressors in addition to *F. psychrophilum* and IHNV exposure are important determinants for fish survival in an aquaculture setting. Here, this was supported by the finding that fish exposure to reuse water for short durations in the pulse and recipient groups had higher survival than those exposed to continuous reuse water. This was despite the detection and development of a closed epidemic of IHNV and *F. psychrophilum* in the recipient treatment after transferring fish exposed to reuse water to these tanks. This indicated that it was not only transmission of pathogens that governed survival. These results highlight the importance of the evaluation of fish performance under production conditions and suggest that the replicated tank system may be useful for high-throughput phenotyping to select for fish that exhibit robust survival in reuse water, as well as further investigation of disease dynamics in the field.

### 4.3 IHNV Vaccination and Reuse Water

IHNV DNA vaccination is highly effective in rainbow trout against immersion challenge (6, 12–14). In this study, both genetic lines were nearly completely protected against static laboratory challenge with single infections of IHNV at a dose of 2 x 10^5^ pfu/ml for 1 h, confirming the high efficacy of the DNA vaccine used in this study (**Supplementary Figure 3A**). Interestingly, a 3-way interaction was observed between genetic line, vaccination and pathogen that was largely attributable to a greater protection of the vaccine in the Tx genetic line when exposed to both pathogens (**Table 6**). Likewise in the field study, vaccination significantly reduced the hazard of mortality (**Table 3**) as well as IHNV loads (**Table 4**). However, eventually vaccine protection was overwhelmed, and all fish died in reuse water in the field, highlighting the importance of presumably other unknown factors such as physiological adaption to low oxygen, additional pathogens, or microbiome changes required for adaption to reuse water. These findings were supported by the laboratory study, wherein vaccine protection was greatly diminished by co-infection between IHNV and *F. psychrophilum.* Interestingly, there was some evidence that the Tx fish line responded better to vaccination than the CSF line. In the field study, the Tx fish line exhibited an increase in the mean day-to-death after vaccination whereas the CSF line did not. Likewise, the Tx line experienced a greater decrease in the hazard of death after vaccination in the co-infection treatment compared to the CSF line. However, this may have just been driven by the fact that sham vaccinated TX fish suffered more mortality overall. It is unclear what mechanisms might allow one fish line to respond better to vaccination than another, and although the effects here were minimal, this is worthy of further investigation particularly in the context of production performance.

### 4.4 Natural Co-Infection and Laboratory Co-Infection

To our knowledge, this study is the first to report IHNV and *F. psychrophilum* co-infection rates following experimental reuse water exposure in an aquaculture relevant environment. Pulsed or constant reuse water was associated with a natural coinfection prevalence of 10% to 45% in fish that had been sham vaccinated. Interestingly, IHNV loads were highest in co-infected fish. Vaccination greatly reduced co-infection by removing IHNV. Others have reported a co-infection effect between *F. psychrophilum* and IHNV where presence of one exacerbates disease caused by the other, as well as pathogen loads. The mechanism(s) driving this phenomenon are unknown but immune suppression has been postulated (11). The lower *F. psychrophilum* external loads in IHNV vaccinated fish observed here highlight the likely importance of IHNV as a primary driver of co-infection. It is possible that IHNV compromises the immune response or the mucosal barrier and this allows *F. psychrophilum* expansion. Furthermore, the high prevalence of *F psychrophilum* overall, particularly in external tissues, would suggest that it is more ubiquitous in the environment than IHNV. It could be that co-infection with IHNV transitions these common, relatively benign *F. psychrophilum* infections, into virulent ones. This is in line with the finding of Ma et al. (11) that mortality was higher when IHNV proceeded *F psychrophilum* infection, compared to vice versa. Regardless of the mechanism, there was strong evidence in our study that co-infection diminished vaccine efficacy, both in the field and laboratory. This could have broad implications for vaccine utility, given the commonality of co-infection in the field, and warrants further investigation.

### 4.5 Study Caveats

In our exposure paradigm, fish were moved by netting and immediately placed in a production environment without an acclimation period. While nylon mesh nets were used for transfer, all netting involves some disruption of skin surfaces and this may expose tissue to elevated pathogen invasion. Infection of skin and fin surfaces by *F. psychrophilum* is known to be enhanced by abrasion and mucus membrane disruption (51–53). Thus, the infection kinetics and prevalence estimates may be upwardly biased. On the other hand, it conceivable that the results may be downwardly biased as the fish density was lower than the production raceways, and they were only exposed to reuse water for 28 days. It should be noted that fish grading and movement in aquaculture settings is often through mechanical means (i.e., pumping, netting and bar grading) and thus periods of elevated abrasions/mucus disruption are common. Another caveat is that we utilized a rapid exposure to reuse water. The typical transition on-farm between first use and fourth use water is gradual occurring over 4-7-month acclimation timeframe. It is possible that a more gradual acclimation to reuse water might induce compensatory changes to physiology or immune system development and thus the morality and pathogen load measurements might be lower in gradually acclimated animals. Nevertheless, these findings represent a baseline for future comparison. Future research efforts should be directed toward unbiased survey of fish-associated microorganisms as well as in-depth analysis of the physiological and immunological state of fish exposed to constant reuse water.

### 4.6 Conclusions

In conclusion, this study underscores the importance of environmental factors, host genetics, and vaccination on infection and mortality. Clearly, exposure to reuse water, containing lower dissolved oxygen content, and likely other factors related to fish production, can lead to increased susceptibility, coinfection and mortality. The replicated outdoor tank system represents a useful model for modulating the timing and duration of exposure and thereby facilitating the study of multi-pathogen infection dynamics. Further characterization of the deleterious factors in reuse water affecting susceptibility is warranted to better understand disease outbreaks and devise effective intervention strategies that are robust to commercial aquaculture conditions.

## Data Availability Statement

The original contributions presented in the study are included in the article/**Supplementary Material**. Further inquiries can be directed to the corresponding authors.

## Ethics Statement

The animal study was reviewed and approved by University of Idaho IACUC and Virginia Institute of Marine Science IACUC. Written informed consent was obtained from the owners for the participation of their animals in this study.

## Author Contributions

AW, GW, and KL, designed the study. AW and JE vaccinated fish, JE and DJ performed field sampling. JE, DJ, AT, BR extracted samples and performed qPCR. AW, DJ, AT and BR performed lab challenge studies. GW, JE, AW, and TL analyzed data. GW and JE wrote the initial draft. All authors contributed to the article and approved the submitted version.

## Funding

This work was supported by the National Institutes of Health EEID [grant number R01GM113233]; and the US Department of Agriculture, Agricultural Research Service [Project number 1930-32000-006 “Integrated Research to Improve On-Farm Animal health in Salmonid Aquaculture”]. The authors alone are responsible for the content and writing of this paper.

## Author Disclaimer

Mention of trade names or commercial products in this publication is solely for the purpose of providing specific information and does not imply recommendation or endorsement by the U.S Department of Agriculture. USDA is an equal opportunity employer.

## Conflict of Interest

The authors declare that the research was conducted in the absence of any commercial or financial relationships that could be construed as a potential conflict of interest.

## Publisher’s Note

All claims expressed in this article are solely those of the authors and do not necessarily represent those of their affiliated organizations, or those of the publisher, the editors and the reviewers. Any product that may be evaluated in this article, or claim that may be made by its manufacturer, is not guaranteed or endorsed by the publisher.

## Supplementary Material

Supplementary Figure 1 Daily mortality (blue line) and cumulative percent mortality (red line) in the 9 raceways that provided the reuse water. The time-course include ~1 week prior to and the duration of the experimental period when CSF and Tx line fish were either pulsed or constantly exposed to reuse water. In each graph, the cumulative percent mortality was reset at the initiation of the experiment (day -1). The complete dataset was not available for raceways 2B and 4B.

Supplementary Figure 2 Image of fish with external discoloration and marbling **(A)** and enlarged spleen **(B)**.

Supplementary Figure 3 Laboratory challenge with viral, bacterial or a combination of both pathogens and is the same data as shown in **Figure 6**. Plots are the cumulative survival of two genetic lines of rainbow trout challenged with either IHNV **(A)**, *F. psychrophilum* **(B)**, or both pathogens simultaneously at same dosages as single infections **(C)**. Genetic lines of rainbow trout, CSF (square symbol) and Tx (circle symbol) were immunized with a DNA vaccine against IHNV (solid line) or sham vaccinated (dotted line) 30 days prior to challenge.
