# Supplementary figures and images for: Aquaculture Reuse Water, Genetic Line, and Vaccination Affect Rainbow Trout (Oncorhynchus mykiss) Disease Susceptibility and Infection Dynamics
Source: Front Immunol. 2021 Sep 22;12:721048. doi: 10.3389/fimmu.2021.721048 (PMC8493035; doi:10.3389/fimmu.2021.721048)

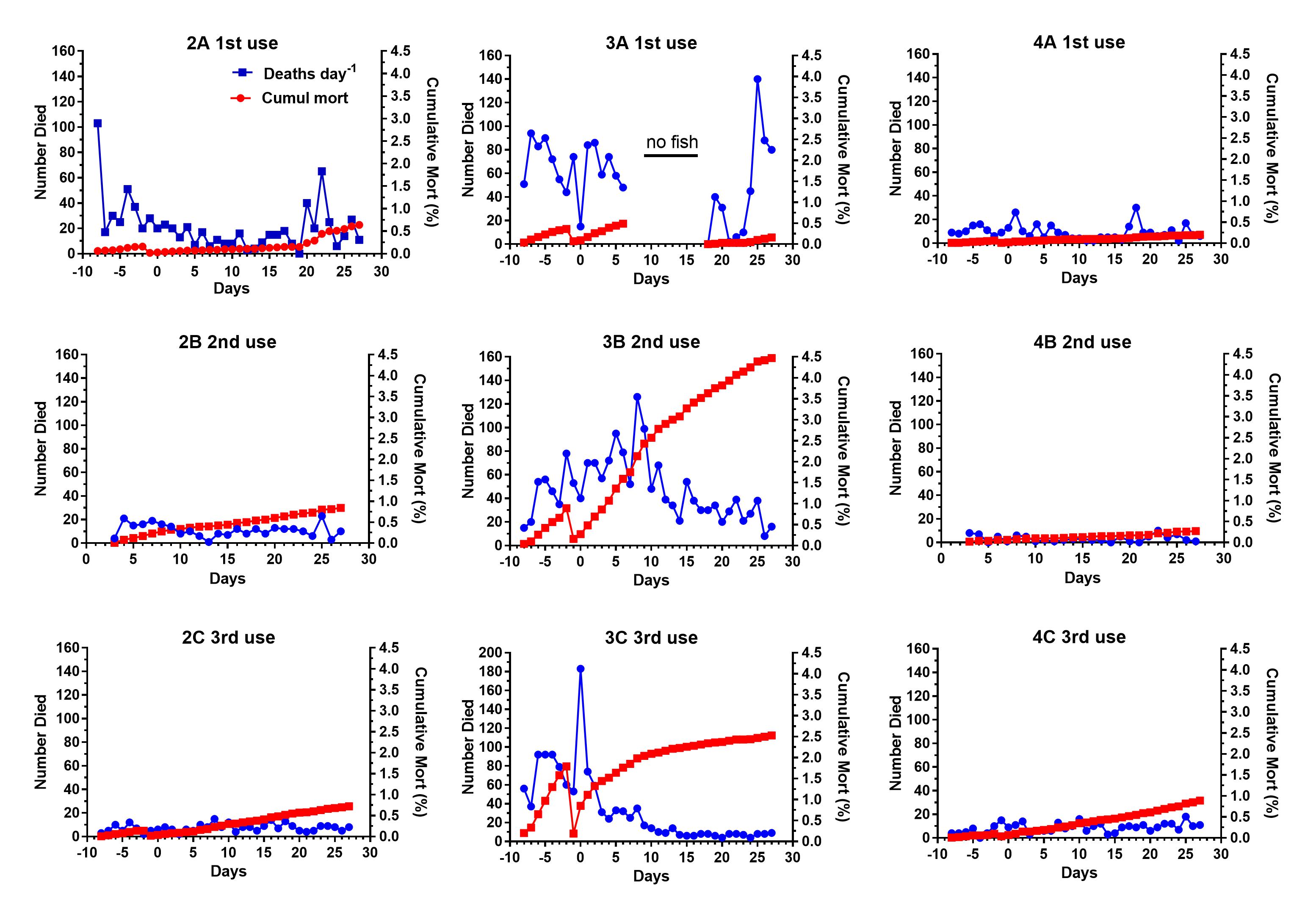

Supplement: Supplementary Figure 1 — Daily mortality (blue line) and cumulative percent mortality (red line) in the 9 raceways that provided the reuse water. The time-course include ~1 week prior to and the duration of the experimental period when CSF and Tx line fish were either pulsed or constantly exposed to reuse water. In each graph, the cumulative percent mortality was reset at the initiation of the experiment (day -1). The complete dataset was not available for raceways 2B and 4B. [file Image_1.jpeg]

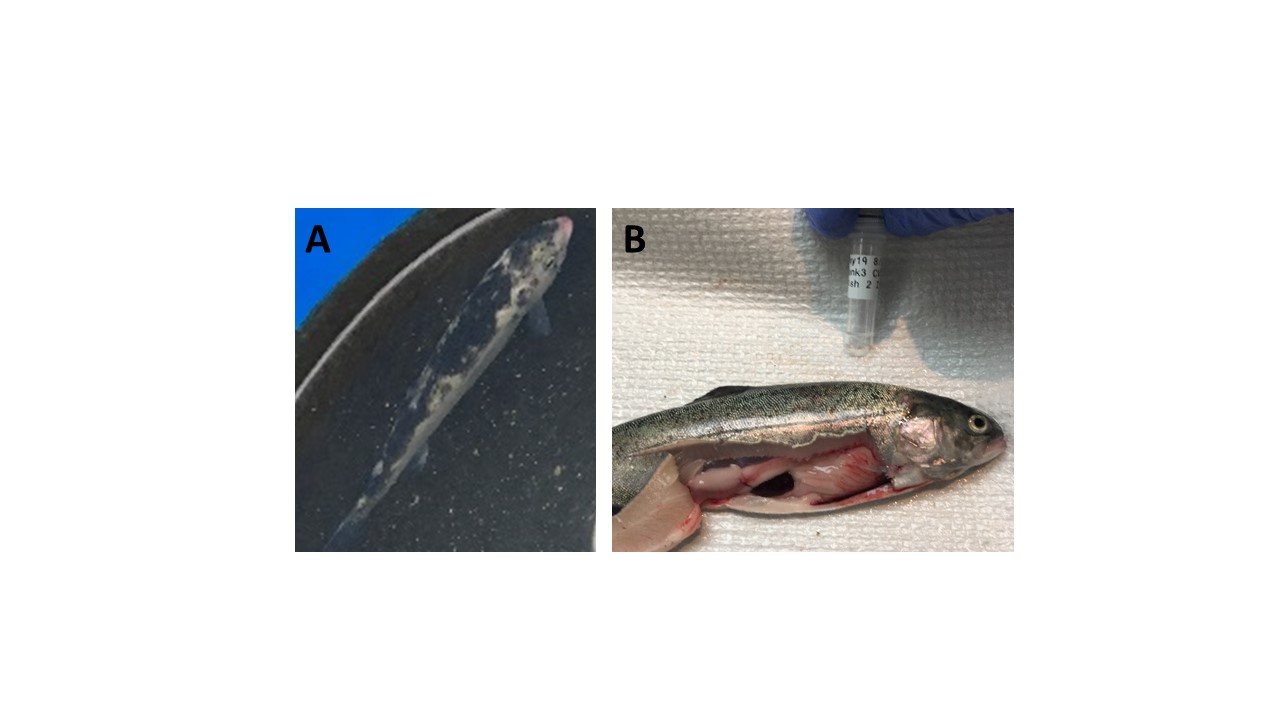

Supplement: Supplementary Figure 2 — Image of fish with external discoloration and marbling (A) and enlarged spleen (B). [file Image_2.jpeg]

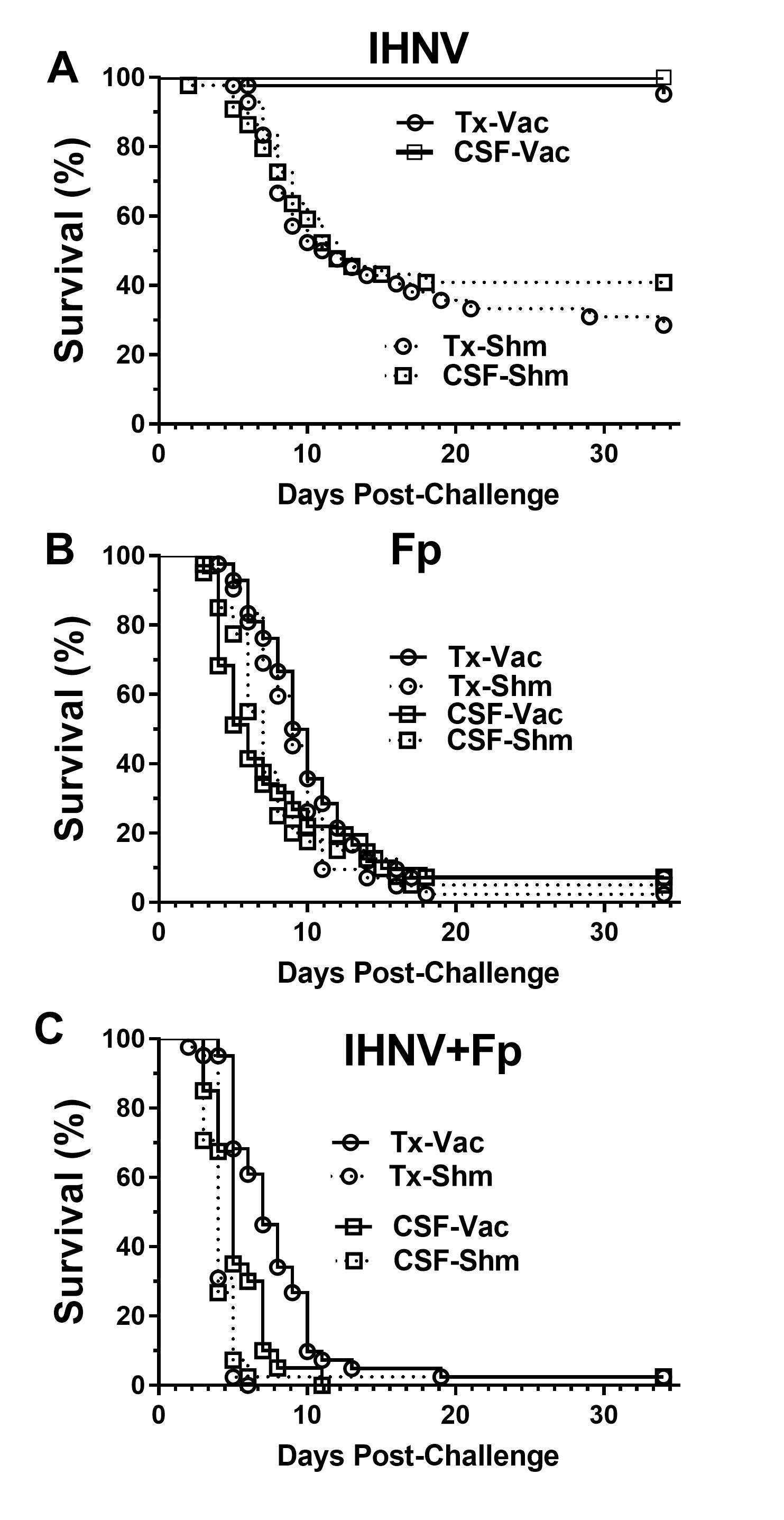

Supplement: Supplementary Figure 3 — Laboratory challenge with viral, bacterial or a combination of both pathogens and is the same data as shown in Figure 6. Plots are the cumulative survival of two genetic lines of rainbow trout challenged with either IHNV (A), F. psychrophilum (B), or both pathogens simultaneously at same dosages as single infections (C). Genetic lines of rainbow trout, CSF (square symbol) and Tx (circle symbol) were immunized with a DNA vaccine against IHNV (solid line) or sham vaccinated (dotted line) 30 days prior to challenge. [file Image_3.jpeg]
